# Balancing Statistical Power and Risk in HIV Cure Clinical Trial Design

**DOI:** 10.1093/infdis/jiac032

**Published:** 2022-02-01

**Authors:** Jillian S Y Lau, Deborah Cromer, Mykola Pinkevych, Sharon R Lewin, Thomas A Rasmussen, James H McMahon, Miles P Davenport

**Affiliations:** Department of Infectious Diseases, Alfred Hospital, Prahran, Australia; Department of Infectious Diseases, Central Clinical School, Monash University, Prahran, Australia; Monash Infectious Diseases, Monash Medical Centre, Clayton, Australia; Infection Analytics Program, Kirby Institute, University of New South Wales, Sydney, Australia; Infection Analytics Program, Kirby Institute, University of New South Wales, Sydney, Australia; Department of Infectious Diseases, Alfred Hospital, Prahran, Australia; Department of Infectious Diseases, University of Melbourne at the Peter Doherty Institute for Infection and Immunity, Melbourne, Australia; Victorian Infectious Diseases Service, Royal Melbourne Hospital at the Peter Doherty Institute for Infection and Immunity, Melbourne, Australia; Department of Infectious Diseases, University of Melbourne at the Peter Doherty Institute for Infection and Immunity, Melbourne, Australia; Department of Infectious Diseases, Alfred Hospital, Prahran, Australia; Department of Infectious Diseases, Central Clinical School, Monash University, Prahran, Australia; Monash Infectious Diseases, Monash Medical Centre, Clayton, Australia; Infection Analytics Program, Kirby Institute, University of New South Wales, Sydney, Australia

**Keywords:** analytical treatment interruption, HIV cure, HIV infection, modelling, posttreatment controllers, viral rebound

## Abstract

**Background:**

Analytical treatment interruptions (ATI) are pauses of antiretroviral therapy (ART) in the context of human immunodeficiency virus (HIV) cure trials. They are the gold standard in determining if interventions being tested can achieve sustained virological control in the absence of ART. However, withholding ART comes with risks and discomforts to trial participant. We used mathematical models to explore how ATI study design can be improved to maximize statistical power, while minimizing risks to participants.

**Methods:**

Using previously observed dynamics of time to viral rebound (TVR) post-ATI, we modelled estimates for optimal sample size, frequency, and ATI duration required to detect a significant difference in the TVR between control and intervention groups. Groups were compared using a log-rank test, and analytical and stochastic techniques.

**Results:**

In placebo-controlled TVR studies, 120 participants are required in each arm to detect 30% difference in frequency of viral reactivation at 80% power. There was little statistical advantage to measuring viral load more frequently than weekly, or interrupting ART beyond 5 weeks in a TVR study.

**Conclusions:**

Current TVR HIV cure studies are underpowered to detect statistically significant changes in frequency of viral reactivation. Alternate study designs can improve the statistical power of ATI trials.

## Teaser

Mathematical modelling was used to show how HIV cure study design can be improved to maximize statistical power, while minimizing risks to participants. Alternate study designs are also explored and discussed in the context of real-world HIV cure study conduct.

Human immunodeficiency virus (HIV) cure-focused research aims to identify strategies or interventions to achieve sustained virological control in the absence of antiretroviral therapy (ART). The gold standard in assessing the efficacy of these interventions is to observe the kinetics of viral rebound when ART is stopped, known as analytical treatment interruption (ATI). Research towards a cure for HIV is increasingly investigating agents not previously used in humans or drugs developed for use in cancer where a higher toxicity rate is accepted. This is reflected in smaller sample sizes in HIV cure interventional studies [1]. Consensus guidelines on how to conduct ATI have been published, which aim to balance ethical and experimental considerations in designing such trials [2]. However, as interventions differ in their mechanism of action and optimal outcome in terms of virological control, there is still no uniform recommendation on which viral load (VL) criteria or end points should be used to trigger recommencement of ART. Moreover, consideration of the statistical power of ATI trials to identify treatment effects, and how trial design affects this, have not been thoroughly addressed.

**Figure 1.**
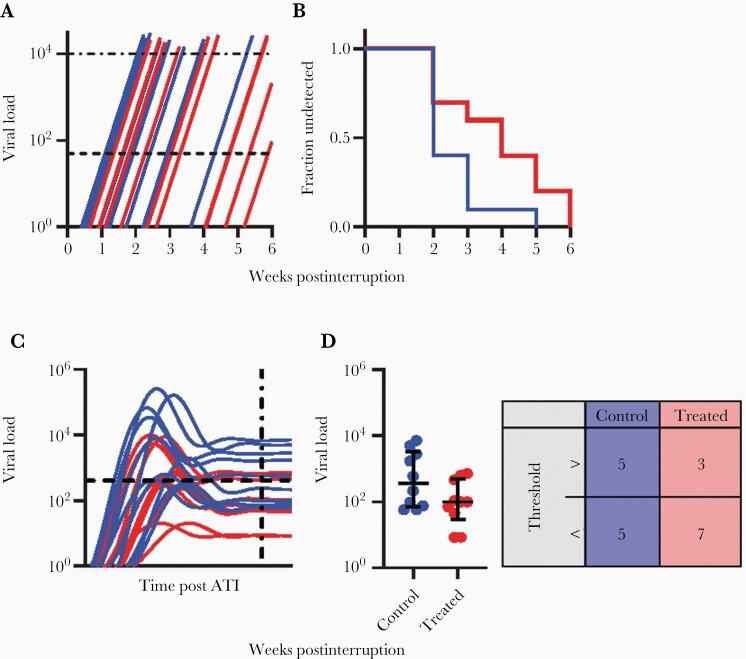
Viral rebound patterns during analytical treatment interruption. *A*, Schematic representation of TVR studies. Participants remain off ART until plasma viral load exceeds a detection threshold (lower dotted line = 50 copies/mL), at which time viral rebound is detected. ART is then initiated once a preset viral threshold level is reached (upper dotted line - assumed to be 10 000 copies/mL). Blue lines, plasma viral load trajectories of a hypothetical control group with usual frequency of viral rebound (average of around once every 7 days after the 1st week). ART is recommenced followed by reduction in plasma VL (not shown). Red line, hypothetical treatment group with a reduced frequency of reactivation from latency, leading to delay in VL rebound. *B*, The time-to-rebound is compared between the groups using survival analysis of time to detection of virus (log rank test). *C*, Schematic of studies of postrebound viral control. Participants remain off ART for a prolonged period during a treatment interruption so that viral loads reach a set-point level. The VL thresholds that trigger restart of ART can be much higher (up to 100 000 copies/mL). Blue line, control group with viral rebound and on average high set-point VL (including some participants with spontaneous viral control). Red line, treatment group with establishment of lower set-point VL; horizontal dotted line shows the median viral load set-point in the control group, measured at the time shown by the vertical dotted line. *D*, Improved postrebound control after treatment can be measured by differences in set-point viral levels (*t* test) or the increased proportion of treated participants maintaining VL less than some predetermined level (Fisher exact test). Abbreviations: ART, antiretroviral therapy; ATI, analytical treatment interruption; TVR, time to viral rebound; VL, viral load.

Individuals who cease ART typically experience viral rebound after 2 to 3 weeks [3]. A delay in viral rebound beyond this in the setting of an interventional study infers efficacy of the intervention in reducing the size of the HIV reservoir and/or enhancing immune control of HIV. Two distinct types of ATI trials have been performed and have different trial end points: time to viral rebound (TVR) studies and set-point studies. TVR studies have delay in viral detection as their primary end point, have lower thresholds to restart treatment, and thus shorter duration of interruption [1]. Studies assessing interventions that may enhance immune control of HIV replication are sometimes designed as set-point studies. These studies allow a period of viremia in the expectation that viral replication may subsequently be controlled at a lower and acceptable level. Set-point studies have longer durations of interruptions and higher VL thresholds to restart ART than TVR studies [1]. While ATI has been safely performed in closely monitored TVR studies [4–6], transmission of HIV [7, 8] and serious adverse events including death from a myocardial infarction [9] have been reported in longer set-point studies. The different types of studies require different statistical approaches to measure treatment effects. Differences in TVR are typically analyzed using a survival analysis approach (such as the log-rank test on time to virus detection), whereas set-point studies may either compare mean VL between groups or the proportion of individuals maintaining VL below a predetermined level (Figure 1).

In designing an interventional trial there are multiple considerations when selecting either a TVR or set-point end point. TVR studies may miss posttreatment controllers due to their shorter duration of interruption and lower VL threshold to restart treatment. However, this briefer interruption period may also be more acceptable for participants, and minimizes the risk of adverse effects and transmission. In TVR studies, statistical power to detect a given effect size can be affected by sample size, frequency of sampling, duration of interruption, or varying VL thresholds to restart treatment following interruption.

Here, we used a modelling approach to examine the impact of these factors on the statistical power to detect differences between intervention and control groups in TVR studies. We also explored whether traditional methods of performing set-point studies could be modified to minimize periods of uncontrolled viremia, while still maintaining statistical power. Finally, we calculated the risk of HIV transmission during ATI, considering the impact of the threshold to restart ART and frequency of VL monitoring. Collectively, our work aimed to use modelling to enhance ATI methodology to achieve an optimal balance between statistical power and participant risk and acceptability.

## METHODS

We used modelling to estimate the (1) number of participants, (2) timing of sampling, and (3) duration to follow-up required to detect a significant difference in the time to detection of virus between control and intervention groups in a TVR study. Models were parameterized based on previously observed dynamics of time to detection of virus following treatment interruption [10]. We compared control and intervention groups using a log-rank test, and performed comparisons using both analytical and stochastic techniques.

To determine the number of participants required to have sufficient power to detect an increase in the overall proportion of posttreatment controllers we compared the expected proportion of detectable controllers between the control and intervention groups. The risk of HIV transmission during viral rebound as a result of ATI was estimated using a previously described stochastic model simulating viral rebound following remission [11]. Full methods are in Supplementary Materials.

## RESULTS

### Sample Size and Statistical Power in Time to Rebound Studies

A major question in ATI studies is how many study participants are required to detect a given effect size of an intervention. Viral rebound is rarely detected in the first week of ATI, but after this period the dynamics of time to viral detection are consistent with an average frequency of successful viral reactivation of around once a week [10, 12]. If the frequency of reactivation decreases (due to a successful intervention), there is an associated increase in the average TVR (Supplementary Figure 1).

**Figure 2.**
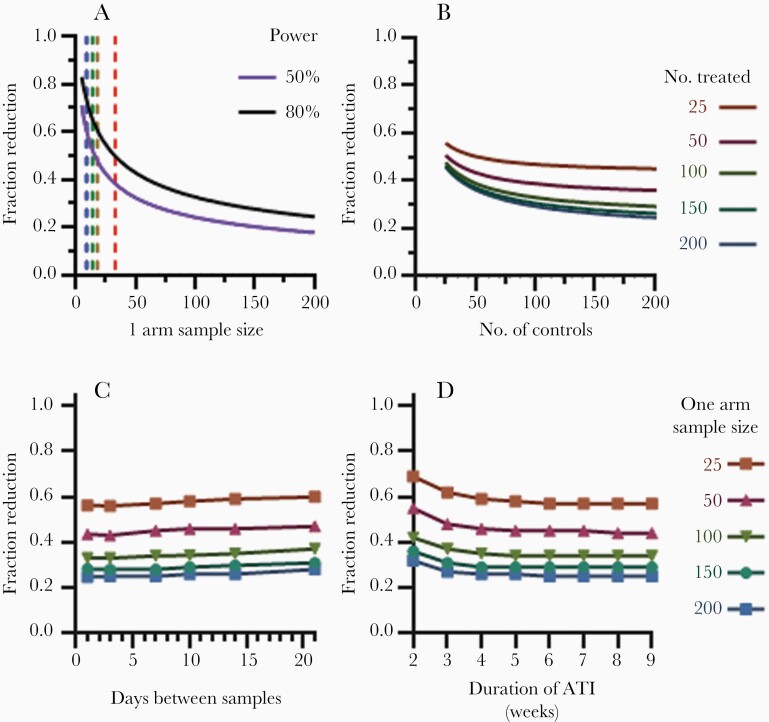
Detectable reduction of the rate of virus reactivation using time-to-detection method in a model of a controlled study. *A*, Number of participants in a single arm needed to detect a given reduction in reactivation rate with power of both 80% (black curve) and 50% (purple curve). Dashed vertical lines show the number of participants in studies previously analyzed [10]: blue, panobinostat study (9 participants in control arm) [13]; green, Swiss-Spanish study (14 participants in control arm) [14]; brown, IL2 study with 18 participants (single arm) [3]; and red, PULSE study (33 participants in control arm) [15]. *B*, Reduction in reactivation rate that could be detected with 80% power, given a treatment group size of 25, 50, 100, 150, and 200 participants and variable number of historical controls. *C* and *D*, The effect of frequency of sampling and duration of ATI on detecting a reduction in reactivation rate with 80% power. We simulated clinical trials with equal sizes of control and treatment arms to detect a difference in time to viral rebound above 50 copies/mL between study arms (using log rank test) given different (*C*) sampling intervals assuming 9-week follow-up and (*D*) durations of ATI assuming weekly VL sampling. Abbreviations: ATI, analytical treatment interruption; VL, viral load.

Figure 2 demonstrates the difference in reactivation frequency that could be detected with studies of different sizes using a traditional power analysis. Historical treatment interruption studies with various sample sizes are shown in Figure 2A. For example, in a study with 13 participants, an intervention would need to induce a 70%–80% reduction in reactivation frequency to have 80% power to detect a delay in TVR and would need to induce a 50%–60% reduction for 50% power to detect a delay. For an 80% power to detect a smaller change in the frequency of successful reactivation (ie, 30% reduction) over 120 participants per arm are required.

### Using Historical Controls in Future ATI Studies

One approach to increasing the statistical power of studies is to enlarge the pool of controls by including historical controls [16, 17]. This might include true controls who received no intervention and individuals who received interventions that did not have any effect on the reactivation frequency. We investigated how accumulating numbers of historical controls would contribute to increased statistical power (Figure 2B).

With increasing number of controls included, we only found a minor increased power to detect changes in reactivation rate. For example, if we had a single study with 50 participants receiving the intervention and 50 control participants, we could detect a reduction in reactivation frequency of greater than 43% with at least 80% statistical power. If 150 historical controls were included (ie, total of 200 controls), we would have 80% power to detect a reduction in reactivation frequency of greater than 36% (ie, 7% gain over a study with no historic controls). This shows that adding historical controls had a limited ability to improve the statistical power of a study (Figure 2B).

### Frequency of Viral Load Monitoring

ATI trial participants need plasma VL and CD4 T-cell counts, as well as clinical reviews on a regular schedule to ensure participant safety and assess study end points. Using modelling, we determined that weekly VL monitoring maintained statistical power to detect differences in TVR with little loss of power compared to more frequent sampling (Figure 2C). For example, in a controlled study with 100 participants in each arm, there would be 80% power to detect a 33% reduction in the frequency of reactivation if VL was measured twice weekly. In contrast, with sampling every 7 or 14 days a 34% or 35% reduction, respectively, could be detected with the same power (Figure 2C). If this study had only 25 participants in each arm, a 56% decrease in reactivation frequency could be detected with twice-weekly sampling, increasing to 59% for fortnightly sampling. This suggests there is little statistical advantage to measuring VL more than weekly, or even fortnightly in settings where weekly monitoring is not feasible. This allows for reasonable frequency of monitoring, taking into consideration participants (and their sexual partners) safety and convenience.

### Duration of ATI

Because of the stochastic nature of viral rebound and the potential for an intervention to delay viral rebound for an unknown duration, some participants may not rebound for many weeks. Prolonged monitoring incurs additional costs for the trial and is undesirable for trial participants. Figure 2D illustrates that with weekly sampling, there is no change in the treatment effect that can be detected at 80% power if the time off ART is extended past 5 weeks. Regardless of the number of participants in each arm, increasing the duration of interruption beyond 5 weeks only increased the detectable reduction in reactivation rate by 1% at most. Therefore, in TVR studies, where the aim is to establish a delay in viral rebound with the intervention, a maximum interruption duration of 5 weeks is appropriate.

**Figure 3.**
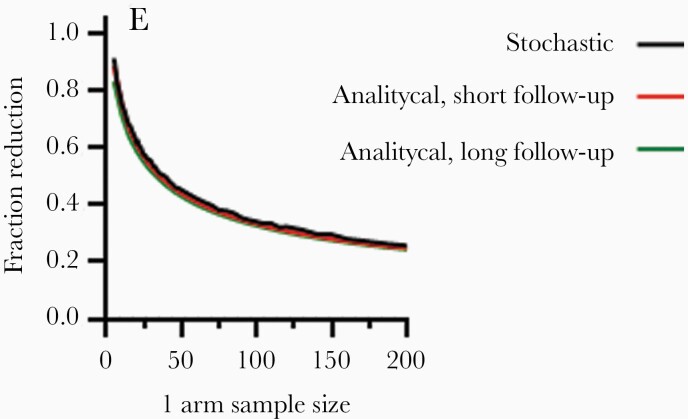
Five-week ATI with weekly VL monitoring similar to indefinite continuous monitoring. Short-course ATI with weekly sampling maintains statistical power (80%) of time-to-detection trials. Green curve shows analytical relationship that assumes continuous detection of reactivation and a long time window (all participants are detected). Red curve shows the analytical relationship corrected for the number of participants that are expected to be detected within 5 weeks of stopping ART. Black curve shows a stochastic simulation assuming weekly sampling and 5 weeks’ ATI. Abbreviations: ART, antiretroviral therapy; ATI, analytical treatment interruption; VL, viral load.

To verify results, the number of participants required to detect reductions in reactivation rate with 80% power was assessed. The gold standard scenario of continuous monitoring for an indefinite period was compared with a stochastic simulation in which participants were sampled weekly for 5 weeks. Figure 3 demonstrates almost identical curves for these 3 scenarios, indicating that a 5-week ATI study with weekly VL monitoring is almost identical in terms of statistical power compared to continuous monitoring for an indefinite period.

### Designing ATI to Detect Posttreatment Control of HIV

The models above examined interventions affecting the frequency of HIV rebound and time to detection of HIV. This is applicable for interventions that may decrease the size or activity of the reservoir. Previous studies have suggested that a subset of individuals may experience good control of plasma viremia off ART, termed posttreatment controllers [18, 19]. This fraction may be increased by early treatment or with interventions attempting to stimulate HIV-specific immune responses [19–21]. Previous ATI studies investigating the degree of posttreatment control (PTC) have typically assessed VL during prolonged treatment interruption [1].

One of the largest studies describing PTC is the CHAMP cohort [18]. This study analyzed viral dynamics post-ATI in over 700 individuals from 14 separate trials identifying 67 posttreatment controllers. Control was defined as having at least two-thirds of virus measurements below 400 copies/mL for up to 24 weeks postinterruption [18]. Rates of PTC were lower if ART was initiated during chronic infection, as compared to acute infection (4% versus 13%) [18]. Using this assumption of a baseline 4% of participants exhibiting PTC, 60 participants would need to be monitored off ART for 24 weeks to have an 80% power to detect a 5-fold increase in PTC from the baseline 4% to 20% (details of calculation given in Supplementary Materials).

**Figure 4.**
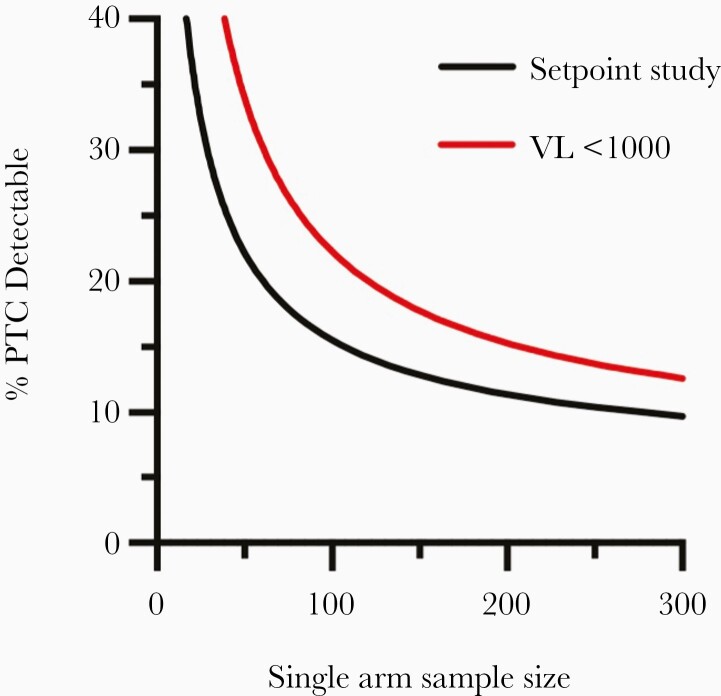
Detecting changes in posttreatment control. Assuming a baseline proportion of posttreatment controllers of 4%, the sample size required to detect an increase in the proportion of controllers at different viral load thresholds is compared to the sample size required for traditional set-point studies summarized by Namazi et al [18]. Abbreviations: PTC, posttreatment controller; VL, viral load.

Namazi et al found that 55% of individuals sampled weekly who met criteria for PTC had early peak VL that remained below 1000 copies/mL, compared to 0% of individuals who did not meet criteria for control [18]. Given the risks of prolonged treatment interruption, the choice of VL threshold for recommencing ART is a balancing act. We used a modelling approach to explore how different VL thresholds for recommencing ART affect the statistical power of an ATI study to detect differences in the proportion of PTC between control and intervention groups (Figure 4). A more conservative approach would be to restart ART when VL exceeded 1000 copies/mL. Namazi et al demonstrated that this would effectively censor up to 45% of controllers due to their early peak in VL exceeding 1000 copies/mL [18].

Assuming then that only 55% of PTC could be detected in each arm, 120 participants in an intervention and control arm would be needed to detect a true increase in the proportion of PTC from 4% up to 20%, with 80% power, as the observed number of PTCs would be much fewer than the actual number.

In a study with 100 participants in a control and intervention arm, a conventional set-point study, with high VL thresholds to restart ART could detect a difference in the frequency of PTC of 16% or higher (4-fold increase from assumed baseline of 4%) with 80% statistical power (Figure 4). Using a threshold of 1000 copies/mL would reduce statistical power to detect PTC but would still be able detect a difference in the frequency of PTC of 22% or higher, while avoiding prolonged uncontrolled viremia.

### A Combined Time to Viral Rebound and Set-Point Study Design

**Figure 5.**
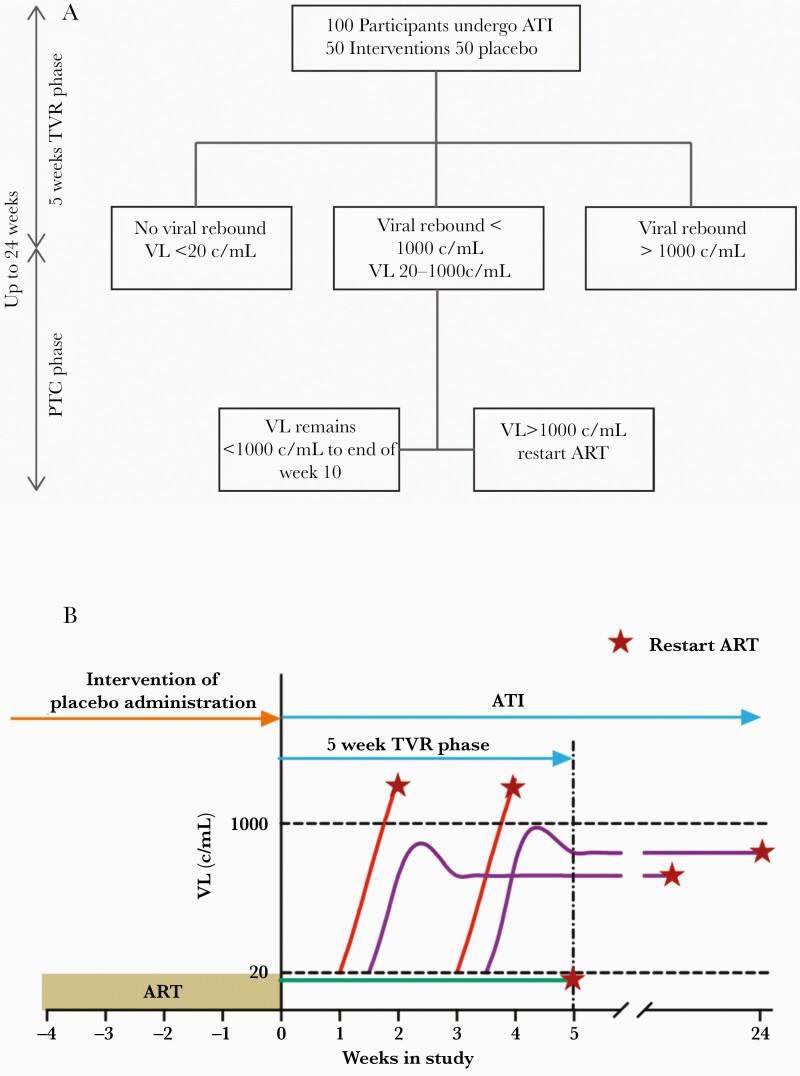
*A* and *B*, Proposed short-course analytical treatment interruption. ART is restarted at any time point if HIV VL > 1000 c/mL (*B* red line). In participants with no VL rebound during the 5 week time to viral rebound phase, ART is restarted at the end of week 5 (*B* green line). Participants with viral rebound between 20 and 1000 c/mL in the time to rebound phase progress to a posttreatment control phase. Participants with VL < 1000 c/mL are termed postrebound controllers (*B* purple line). Abbreviations: ART, antiretroviral therapy; ATI, analytical treatment interruption; c/mL, copies/mL; PTC, posttreatment controller; TVR, time to viral rebound; VL, viral load.

An alternative to the prolonged ATI required to identify PTC in set-point studies is to look for a signal in viral rebound kinetics to predict individuals who may later achieve virological control, based on observations in previous PTC cohorts. We demonstrate an alternate study design where a 5-week TVR study is followed by a modified set-point study to determine the proportion of PTC (Figure 5A and 5B). Individuals who had viral rebound to detectable levels but remained below 1000 copies/mL would continue to be closely monitored off ART for up to 24 weeks. If VL exceeds 1000 copies/mL at any point in this study, ART would be restarted so participants would avoid prolonged uncontrolled viremia.

End points measured include proportion of participants with no virus rebound, a survival curve in the TVR phase, or proportion of controllers in the set-point phase. Observing the proportion of posttreatment controllers in these studies could identify an effective intervention and justify a subsequent open-ended ATI trial.

## Estimating the Risk of Transmission During ATI

**Figure 6.**
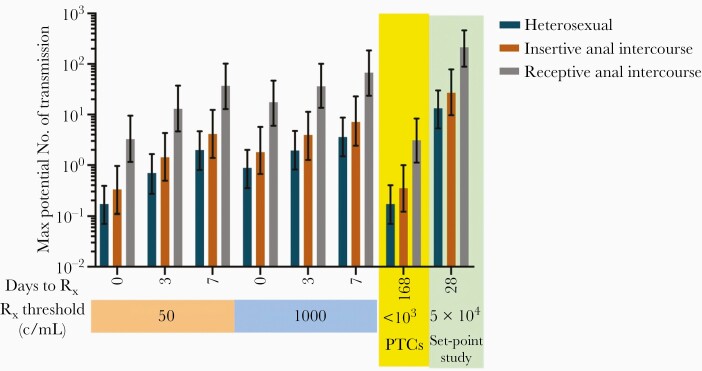
Predicting the maximal risk of transmission for different study designs: Using a stochastic modelling approach (described in [22]) to predict time of reactivation, virus growth, and risk of HIV transmission per 1000 participants (in the absence of counselling and behavioral change) for different study designs and routes of transmission (Error bars show 95% uncertainty intervals of the estimates). The delay between virus detection and commencement of treatment is a major factor determining transmission risk. Extending studies to follow PTCs for an additional 24 weeks has minimal effect (yellow shading). These are contrasted to predicted transmission in a previous set-point study (green shading) [23]. Note the log scale on the y-axis. Abbreviations: c/mL, copies/mL; PTC, posttreatment controller; Rx, treatment.

For short-course (5 week) time to detection studies with weekly monitoring, the risk of transmission was 2 per 1000 participants (95% uncertainty interval [UI], 0.8–4.7) for heterosexual intercourse if ART was recommenced at plasma HIV RNA at 50 copies/mL and treated at their next (weekly) visit (Figure 6). This rose to 3.6 per 1000 participants (95% UI, 1.5–8.7) if the threshold to restart ART was 1000 copies/mL. These transmission numbers effectively doubled for insertive anal intercourse, and increased nearly 20-fold for receptive anal intercourse (Figure 6). Risk of transmission can be decreased dramatically if point of care VL testing is available at weekly visits and ART is given immediately (reduced to 0.2 and 0.9/1000 participants for heterosexual intercourse if ART is recommenced at viral detection and VL > 1000 copies/mL, respectively).

Set-point studies inevitably incorporate a longer exposure to virus for some participants. We calculated the risk of transmission (combining control and treatment arms) in a recent clinical set-point trial, to be 13 heterosexual transmissions per 1000 participants (95% UI, 5.3–30). This could have been as high as 214 transmissions per 1000 participants (95% UI, 89–460) for receptive anal intercourse [22]. Note, these estimates of transmission risk are for unprotected sexual contact and where strategies to minimize HIV transmission are not employed. In our proposed alternative design to detect posttreatment controllers, the additional risk of heterosexual transmission in a control group is under 0.2/1000 participants (95% UI, 0.07–0.4) if the VL remains below 1000 copies/mL for up to 24 weeks. This risk would be higher for insertive anal (0.35/1000; 95% UI, 0.12–1) or receptive (3.1/1000; 95% UI, 1.1–8.3) anal intercourse, but is still much lower than in traditional set-point studies.

## DISCUSSION

In this study, we used mathematical modelling and VL data accrued from historical ATI studies to model viral rebound upon ceasing ART and described the optimal study design to detect a difference in the time to virus rebound between a control and intervention group. We found that an optimally powered TVR study required many more participants than in recently reported ATI trials, and would maintain power with weekly VL monitoring during ATI lasting no longer than 5 weeks (summarized in Table 1).

**Table 1.** Summary of Findings From Modelling of Analytical Treatment Interruption Trial Design Parameters

Variable	n Required for 80% Power	% Reduction in Rebound Frequency^c^	Summary
Sample size	120 (1 arm)	30%	Most recent ATI studies are not large enough to detect reduction in the frequency of viral rebound with high power
Using historical controls	If noncontrolled intervention arm n = 50, 25 historical controls required	…	Using historical controls instead of placebo arms would minimize the number of participants required to test the intervention If only a small number of participants were recruited, a larger sample of historical controls could be used as a comparison while maintaining high power
	If noncontrolled intervention arm n = 25, 50 historical controls required		
Frequency of sampling^a^	…	33% with twice weekly sampling 34% with weekly sampling	There is minimal advantage in measuring VL more frequently than weekly
Duration of ATI^b^	…	38% in 5-week ATI 38% in 9-week ATI	There is minimal advantage to increasing the duration of ATI beyond 5 weeks

Abbreviations: ATI, analytical treatment interruption; n, number of participants; VL, viral load.

Assuming 9-week follow-up.

Assuming weekly VL sampling.

At 80% power with 100 participants in each arm.

Contemporary ART leads to rapid reduction in VL [23], and reduces morbidity and mortality to the point where people with HIV in many countries experience a normal life expectancy [24]. It is therefore crucial that research aimed at achieving a cure for HIV is conducted in a safe and careful manner. Our findings indicate that current ATI studies are only powered to identify interventions that lead to large reductions in the frequency of viral rebound and subsequent long delays to detection of viral rebound. These small proof-of-concept studies are helpful to explore mechanistic effects, but our modelling reveals that most studies are underpowered to detect relevant delays in viral rebound. Future ATI studies will need to find a rational approach to balancing the high sample size requirements to detect a reasonably expected signal, against the risk of overseeing a true effect due to lack of statistical power, whilst also appreciating the accumulated risk associated with ATI in large cohorts.

ATI trials may be more acceptable to prospective trial participants if concerns around transmitting virus were able to be addressed more accurately [25]. We show that the theoretical risk of transmission using various ATI models can be calculated, and this allows for clearer discussions around this with trial participants. While we have measured a numerical risk that may be helpful in consent discussions, it is important to emphasize that there have been no linked sexual transmissions in multiple large cohorts of serodiscordant couples where the HIV-positive partner had a VL < 200 copies/mL [26–28]. Counselling around safe sexual practices and the provision of preexposure prophylaxis is also now recommended for prospective ATI trials [2, 29]. Our work highlights the importance of marrying calculatable risk and robust clinical evidence to optimize the consent process.

Finally, we present the case for a hybrid ATI model that combines a 5-week TVR phase with a 24-week set-point phase, where ART is restarted as soon as VL exceeds 1000 copies/mL. The risk of transmission and other adverse events linked to uncontrolled viremia would be reduced, while still allowing analysis of virological and immunological correlates of prolonged virological control. Our proposed hybrid ATI design allows investigators to determine if there is a signal for the intervention towards delayed TVR, while also potentially identifying PTC in the extended 24-week ATI. The hybrid design would also allow for patients exhibiting delayed TVR or PTC to electively remain off ART if the participants and investigators both agree.

Well-designed clinical trials use control arms to control for longitudinal variation and potential unmeasured confounding factors. Single-arm observational ATI trials are unable to compare other secondary end points (including markers of reservoir, immune response to intervention) and compare any adverse effect/toxicity of an intervention to a control arm. Given their exploratory nature, many recent HIV cure-focused studies have no control arms [1] making it difficult to definitively quantify the impact of the tested intervention, particularly as it is not entirely clear what the true rate of natural PTC is [30]. Conversely, the ethics of interrupting ART only to receive a placebo intervention has been extensively debated [31, 32]. There is a tendency for researchers designing these trials to avoid large sample sizes and control arms to minimize risks of ATI and costs, as well as risks from exposure to the intervention itself. However, this comes at the expense of statistical power to detect an effect of the intervention. Our work has demonstrated that for studies to have adequate statistical power to detect smaller treatment effects, very large sample sizes are required, which does not reflect the status of ATI trials currently being conducted. International research collaborations are needed to enroll participants at multiple sites and expedite recruitment. Historical controls offer another solution to enrolling appropriate participant numbers.

The use of historical controls has many potential advantages; it avoids interrupting ART in people who receive no interventions and aids recruitment and retention to trials where the possibility of being in a placebo arm may deter potential participants. The accumulation of data from individuals who have undergone interruption without an intervention or received an intervention that has no effect could provide a large enough dataset to perform power calculations for large ATI trials and also data on subgroups such as early versus chronic infection.

However, using historical control data is limited by potential confounders when comparing historical with present-day populations. These include historical groups that are more likely to commence ART in chronic HIV infection, compared to the present day where ART is recommended at diagnosis and earlier in the course of infection, typically within 6 months of acquiring HIV [33]. Comparisons between these 2 groups may not be appropriate for some trial designs. Another example is the use of different ART regimens over time, with more potent integrase inhibitors being the current standard of care, which may have different impacts on reservoir size compared to nonintegrase-containing regimens.

ATI is increasingly included in HIV-cure–focused intervention trials, but there are ongoing questions about optimal trial design to detect true effects of reservoir reduction and/or immune control of virus. We have used modelling approaches to propose strategies in ATI trial design that enhance statistical power whilst minimizing risks, as well as allowing more effective counselling of individuals enrolling into ATI studies. With appropriate sample sizes and frequent VL monitoring, TVR studies could be shortened. Early viral rebound kinetics and frequency of PTC in set-point studies could be used to predict performance of the intervention in a subsequent open-ended ATI. Finally, access to deidentified datasets from completed interruption studies could allow for an historical control database, which could aid in the design of future ATI studies providing alternatives to placebo-controlled trials.

## Supplementary Data

Supplementary materials are available at *The Journal of Infectious Diseases* online. Supplementary materials consist of data provided by the author that are published to benefit the reader. The posted materials are not copyedited. The contents of all supplementary data are the sole responsibility of the authors. Questions or messages regarding errors should be addressed to the author.

jiac032_suppl_Supplementary_Materials

## Notes

***Financial support.*** This work was supported by the National Health and Medical Research Council (NHMRC) of Australia (grant number APP1149990 to M. P. D. and S. R. L.); and an National Health and Medical Research Council-European Union collaborative award (grant number APP1115828 to M. P. D.). M. P. D. and D. C. are recipients of NHMRC Investigator awards (grant number 1173528). J. S. Y. L. is a postgraduate fellow (grant number 1151607) and S. R. L. is a practitioner fellow (grant number 1135851) of the NHMRC.

***Potential conflicts of interest.*** J. S. Y. L. and T. A. R. received honorarium from Gilead for speakers’ fees. S. R. L. receives consulting fees from Abivax, Geovax, ViiV, Tetralogic, Vaxxinity, Esfam, and Immunocore. M. P. D. receives an honorarium from eLife for duties as a senior editor. The institutions of J. H. M., J. S. Y. L., S. R. L., and T. A. R. have received grant funding from Merck, ViiV, and Gilead for the conduct of clinical trials. All other authors declare no conflicts of interest.
