# Supplementary material for: Balancing Statistical Power and Risk in HIV Cure Clinical Trial Design
Source: J Infect Dis. 2022 Feb 1;226(2):236–45. doi: 10.1093/infdis/jiac032 (PMC9400422; doi:10.1093/infdis/jiac032)
Supplement: jiac032_suppl_Supplementary_Materials [file jiac032_suppl_supplementary_materials.docx]

**Supplementary Methods**

**Relationship between frequency of reactivation and viral rebound.**

The change in the average time to reactivation varies as the fraction reduction in reactivation rate changes. This relationship is summarised in Supplementary figure 1. As the reactivation frequency is decreased by larger amounts, there is an associated fold increase in the average time to viral reactivation.


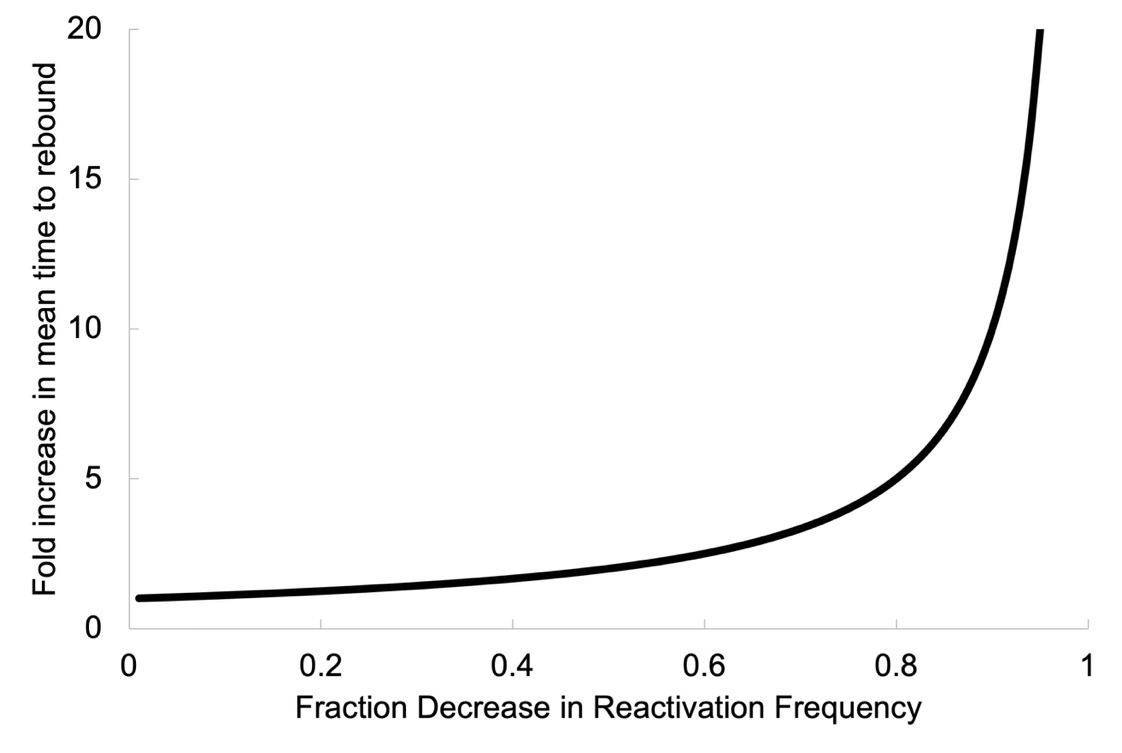


**Supplementary figure 1.** The relationship between time to viral rebound and viral reactivation rate.

**Analytical estimation of statistical power.**

To determine the sample size needed to detect a statistically significant difference in time to detection of virus in blood between control and intervention groups by a log-rank test, we used the following formula from the literature (1):

$n=\frac{(z_{\alpha/2}+z_{\beta})^{2}}{p\pi(1-\pi)\Theta^{2}}$ .

Where *n* is the number of participants in both arms, *π* is the fraction of participants to be allocated to the control group, *z_α/2_* and *z_β_* are quantiles of the standard normal distribution for significance level α and power 1-*β*.

If the time to viral rebound has an exponential distribution with rate $\lambda$ in the control group and rate $(1-f)\lambda$ in the treated ($f$ is the fraction reduction, $f<1)$ then the log-hazard ratio $\theta$, is given by $\theta=1-f$. The value *p* is the probability that we will detect virus in the participant during follow up window (duration of study) *w*. We should note that the detection of virus can only occur after the fixed delay (which is the same for all groups, see the detailed explanation in the stochastic simulation section) so to calculate the probability of event in the follow-up window, *w*, we should subtract 7 days of fixed delay from the value of *w*.


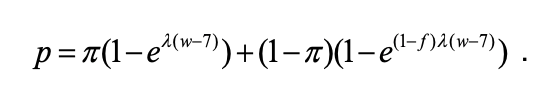
 (2)

Since, in the study we are interested in the fraction reduction *f* as a function of the sample size (*n*), we solved equation 2 numerically with respect to *f* using function NSolve in Mathematica software (Wolfram Research, Champaign, ILL.).

**Estimation of statistical power using stochastic simulation.**

The above asymptotic formula did not allow investigation of the impact of timing of sampling on the statistical power to detect an effect of treatment. In order to investigate the impact of variation of time between sampling and duration of follow-up with a discreet sampling protocol, we developed a stochastic simulation of a time to viral detection study.

We assume, that the time-to-detection of virus after ATI consists of a fixed time interval and the stochastic delay. Fixed time interval incorporated the time needed for ART levels to drop below their inhibitory concentration and the time needed for virus to grow from its initial appearance to the detection threshold (2). We assumed that these two components were the same for all participants and together take 7 days. The time to first rebound that will grow to the detectable level is assumed stochastic, distributed exponentially with mean time of 7 days(3) .

Although the results of simulations presented in this study used this average frequency of once a week, we noted that for different baseline frequencies of reactivation, consistent conclusions would be reached by rescaling all time-dependent variables such as sampling timing, duration of treatment and reactivation rate.

Assumptions of modelling are summarised in **Supplementary figure 2**.

Minimal time

to

successful

reactivation

Possible time

of successful

reactivation

Washout

time

**Log VL**

Minimal time to detection

Time to detection

Possible time of detection

*Detection threshold*

**ART**

**% undetected**

**time**

**time**

**A**

**B**

Time to reactivation

**Supplementary figure 2:** **The assumptions of modelling.**

A) Random rebound and detection of virus in the participant after ATI.

B) Components of the time-to-detection curve that correspond to the within host processes in A.

We assume that reactivation from latency occurs with the same frequency in all participants within the group, but while the drug concentration after ART interruption is high enough (green area) the virus (red solid lines) cannot grow to the detection threshold. We call this period the washout time, which is assumed to be the same for all participants. After the washout time, any successful reactivations also require some time to grow to the detection threshold (growth time), so no positive readings are possible for duration of the washout time + growth time (approximately a week). This leads to a shoulder in the time to detection curve (green and orange parts of time to detection curve respectively). The exponential part of the time-to-detection curve (red solid line) represents the distribution of time to detection (dashed blue line) in different participants in the group.

Using this model, we simulated a random time to detection of virus by generating random times to reactivation and adding a fixed delay as described above. The times when the virus crossed the detection threshold were aligned to the next closest sampling timepoint in order to mimic discrete viral load sampling.

Based on the number of simulated patients with positive viral loads at each sampling timepoint in a given simulation, the model generates the Kaplan-Meier estimate of the distribution of times to detection. Participants with a detection time beyond the duration of study (follow up window) are considered as censored.

Performing 3000 simulations, for each set of parameters and comparing control and treated groups by the log-rank test we estimated the power to detect a difference in the time-to-detection between intervention and control groups (increasing the number of simulations did not significantly reduce the variation of estimated power).

By changing the sample size and fraction reduction by small steps and repeating the procedure described above we obtained a set of vectors with 3 components – power, sample size and fraction reduction. We then selected vectors where power was approximately 80%, thus we obtained the relationship between fraction reduction and sample size with a power of 80%.

The stochastic model was implemented in Mathematica software (Wolfram Research, Champaign, ILL.).

**Determining the number of participants required to detect an increase in post treatment control**

To determine the number of participants, *n,* required in a treatment arm to detect an increase in the overall proportion of post treatment controllers (PTCs), we compared the expected proportion of detectable PTCs without an intervention, $p_{1},$ (taken from (4)) with the expected detectable proportion following an intervention that increased the ratio of PTCs in the population, $p_{2}$. We then used the formula in equation 3 below (taken from p89 of (5)) to calculate *n* as:

$n=\left( z_{\frac{\alpha}{2}}+z_{\beta} \right)^{2}{\frac{\left( p_{1}\left( 1-p_{1} \right)+p_{2}(1-p_{2}) \right)}{\left( p_{1}-p_{2} \right)^{2}}}$ (3)

Where, as above, *z_α/2_* and *z_β_* are quantiles of the standard normal distribution for significance level α and power 1-*β*.

**Estimation of Transmission Risk**

The risk of HIV transmission during ATI was estimated using a stochastic model previously described in (6). We simulated 100,000 participants who were assumed to be taken off ART and monitored weekly for viral rebound. It was assumed that ART was re-initiated when viral loads were detected at greater than a pre-determined threshold (either 50 copies /ml or 1,000 copies/ml in our simulations). The time between viral load detection above the threshold and treatment re-initiation was allowed to vary between 0 days (immediate treatment) and 7 days. The risk of transmission was determined based on the probability of transmission at the viral loads attained between rebound and the time a simulated participant was placed on treatment. Transmission risk was assumed to be zero once the simulated participant restarted ART. Previously published estimates of transmission risk for varying viral loads and routes of transmission (in sero-discordant couples not on HIV pre-exposure prophylaxis (PrEP)) were used to parameterise the model. Full details can be found in (6).

It is possible to estimate the (maximal) transmission risk, based on previous studies in untreated HIV (7-9). We calculated how viral load sampling interval and duration of detectable virus above thresholds where transmission might be possible, affect the risk of transmission using a previously published model of viral rebound and transmission (10). Total risk was calculated using risk per sexual intercourse and also number of acts of intercourse per month.

**References**

1. Hosmer DW, Lemeshow S, May S. Applied survival analysis : regression modeling of time-to-event data. 2nd ed. Hoboken, N.J.: Wiley-Interscience; 2008. xiii, 392 p. p.

2. Pinkevych M, Fennessey CM, Cromer D, Tolstrup M, Sogaard OS, Rasmussen TA, et al. Estimating Initial Viral Levels during Simian Immunodeficiency Virus/Human Immunodeficiency Virus Reactivation from Latency. J Virol. 2018;92(2).

3. Pinkevych M, Cromer D, Tolstrup M, Grimm AJ, Cooper DA, Lewin SR, et al. HIV Reactivation from Latency after Treatment Interruption Occurs on Average Every 5-8 Days--Implications for HIV Remission. PLoS Pathog. 2015;11(7):e1005000.

4. Namazi G, Fajnzylber JM, Aga E, Bosch R, Acosta EP, Sharaf R, et al. The Control of HIV after Antiretroviral Medication Pause (CHAMP) study: post-treatment controllers identified from 14 clinical studies. J Infect Dis. 2018.

5. Chow S-C, Show J, Wang H. Sample Size Calculations in Clinical Research. Chow S-C, editor. Boca Raton, FL, USA: Taylor & Francis Group; 2008. 449 p.

6. Cromer D, Pinkevych M, Rasmussen TA, Lewin SR, Kent SJ, Davenport MP. Modeling of Antilatency Treatment in HIV: What Is the Optimal Duration of Antiretroviral Therapy-Free HIV Remission? J Virol. 2017;91(24).

7. Gray RH, Wawer MJ, Brookmeyer R, Sewankambo NK, Serwadda D, Wabwire-Mangen F, et al. Probability of HIV-1 transmission per coital act in monogamous, heterosexual, HIV-1-discordant couples in Rakai, Uganda. Lancet. 2001;357(9263):1149-53.

8. Boily MC, Baggaley RF, Wang L, Masse B, White RG, Hayes RJ, et al. Heterosexual risk of HIV-1 infection per sexual act: systematic review and meta-analysis of observational studies. Lancet Infect Dis. 2009;9(2):118-29.

9. Jin F, Jansson J, Law M, Prestage GP, Zablotska I, Imrie JC, et al. Per-contact probability of HIV transmission in homosexual men in Sydney in the era of HAART. AIDS. 2010;24(6):907-13.

10. Cromer D, Grimm AJ, Schlub TE, Mak J, Davenport MP. Estimating the in-vivo HIV template switching and recombination rate. AIDS (London, England). 2016;30(2):185-92.
